# EU-Forest, a high-resolution tree occurrence dataset for Europe

**DOI:** 10.1038/sdata.2016.123

**Published:** 2017-01-05

**Authors:** Achille Mauri, Giovanni Strona, Jesús San-Miguel-Ayanz

**Affiliations:** 1 European Commission, Joint Research Centre, Directorate D—Sustainable Resources, Bio-Economy Unit, Via Enrico Fermi 2749, Ispra 21027, Italy

**Keywords:** Plant ecology, Ecology, Climate change, Forestry, Palaeoclimate

## Abstract

We present EU-Forest, a dataset that integrates and extends by almost one order of magnitude the publicly available information on European tree species distribution. The core of our dataset (~96% of the occurrence records) came from an unpublished, large database harmonising forest plot surveys from National Forest Inventories on an INSPIRE-compliant 1 km×1 km grid. These new data can potentially benefit several disciplines, including forestry, biodiversity conservation, palaeoecology, plant ecology, the bioeconomy, and pest management.

## Background & Summary

Forests play a key role in biodiversity conservation, by providing a habitat for the majority of world’s terrestrial animal and plant species^1,2^. Although this is particularly evident in tropical environments, it is also true for temperate habitats^3–6^. In Europe, forests cover approximately 33% of total land area, and their spatial extent has even increased over the past 25 years as a result of land abandonment following growing urbanisation^7,8^. Furthermore, the distribution of forested areas and their species composition is rapidly changing as a consequence of climate change^9^, anthropogenic activities^10^, and increasing natural hazards such as forest fires and windstorms^11^. In this context, satellite data can provide valuable information by detecting forest location and extent^12^, but can do little to map tree species distribution with the appropriate spatial and taxonomic resolution needed to monitor changes in terms of tree community composition and structure.

To tackle this issue, the Joint Research Centre (JRC) of the European Commission supported projects that aim to harmonise European forest information, and coordinated the implementation of European Union (EU) actions regarding forest monitoring, including the Forest Focus Regulation^13^. Hence, the Forest Focus monitoring database (hereafter, the ‘Forest Focus’)^14^ was set up to monitor the effects of atmospheric pollution on forests. In the same framework, a supplementary pilot study, the Biosoil project^15^, was also set up to integrate information about forest soils and forest biodiversity at the European level.

Although the tree occurrence data resulting from the Forest Focus database and the Biosoil project have proved useful in various studies dealing with forestry, climate change, biodiversity and ecosystem services^16–24^, they have significant limitations in terms of spatial data density, spatial resolution and geographical coverage. Furthermore, the methodological protocols for data acquisition in Forest Focus, which were established in the 1980s under Council Regulation EEC No. 3528/86 (ref. 25), lack detail, which possibly affects the reliability and spatial accuracy of the resulting occurrence data.

Another valuable source of forest data for Europe is the Atlas Florae Europeae^26,27^, which provides an exhaustive inventory of vascular plants at the continental scale, and which has been extensively used to estimate large-scale tree species distribution and tree species richness^28–32^. However, the low spatial resolution of the dataset (nearly 50 km) has often limited its applicability, especially in conservation studies, forcing users to manipulate the data by applying downscaling techniques, which can potentially affect the reliability of results^33^.

Additionally, most European countries have implemented forest monitoring and inventory programmes at the national level (the National Forest Inventories, NFIs). The data collected in the context of these programmes have huge potential, both in terms of quantity and quality. However, this important resource has long remained unexploited due to country-specific restrictions on their availability, and to fundamental differences in the national survey procedures, which complicate data harmonisation. To overcome these problems and make NFI data publicly available and readily usable, in 2008 the JRC established a framework contract with European countries (including EU Member States and neighbouring countries) to regulate the provision of forest data and services in support to the European Forest Data Centre (EFDAC)^34^. The motivation behind the framework contract was to provide EU decision-makers with high-quality harmonised forest data to develop and implement environmental policies at European level^35,36^. This eventually led to the compilation of a comprehensive dataset that includes tree occurrence data from 21 countries’ NFIs.

**Figure 1:**
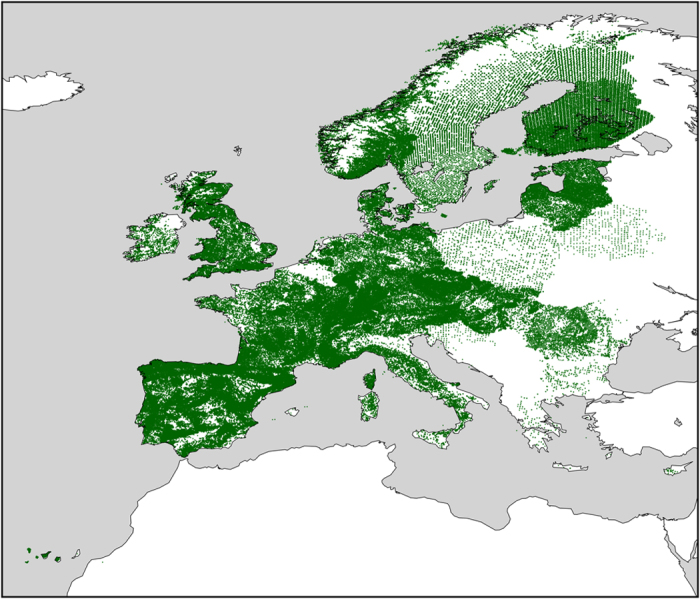
Spatial distribution of all the occurrences present in EU-Forest.

**Figure 2:**
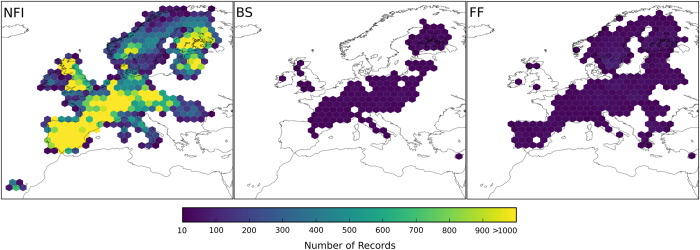
Geographical coverage and density of occurrence records for the three different sources used to compile the dataset. BS, Biosoil; FF, Forest Focus monitoring database; NFI, National Forest Inventories dataset. The colour of each hexagon indicates the corresponding number of records. Hexagons were drawn only if they overlapped with at least 10 occurrences.

We here make those data publicly available, by harmonising and merging them with the tree occurrence data provided by the Forest Focus and Biosoil datasets. The joint dataset, called EU-Forest, has a very high data density (including more than half a million occurrences) and a moderately high resolution of 1 square kilometre (Figs 1 and 2), and is by far the largest collection of tree species occurrences in Europe.

The EU-Forest dataset is an extremely valuable resource for ecological and conservation studies^37^. For instance, detailed data on large-scale tree species distribution may help orient conservation efforts by developing accurate biodiversity indicators^38^, and improve our understanding of how European forests will respond to climate change^39,40^. Furthermore, considering the importance of wood to the European economy^18,41^, the information within the dataset will no doubt have strong political and social implications, possibly improving the international transparency on the geo-political distribution of wood resources.

**Figure 3:**
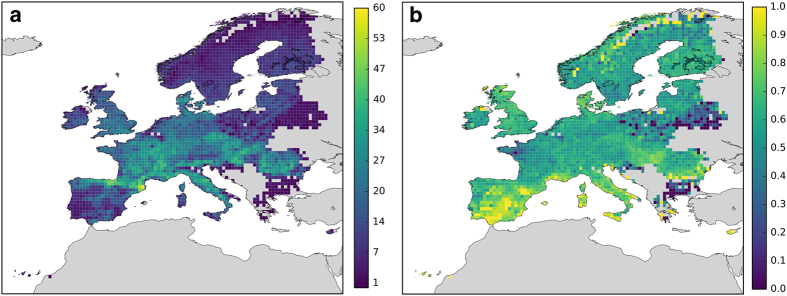
Tree diversity indices. Tree species richness (**a**) and relative rarity (**b**) estimated from the merged dataset at a resolution of 0.5×0.5 degrees. Relative rarity was computed following Leroy *et al.*^68^.

**Figure 4:**
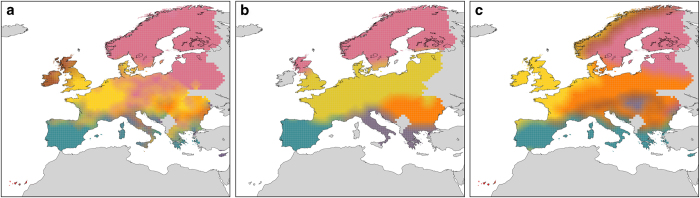
Biogeographical regions. Comparison between the biogeographical classification obtained from our merged dataset using the procedure by Vilhena and Antonelli^59^ at a resolution of 0.5×0.5 degrees (**a**), the classification by Rueda *et al.*^61^ (upscaled to the same resolution to ease comparison, **b**), and the European classification of biogeographical regions (**c**).

The EU-Forest dataset also has the potential to improve our preparedness with regard to forest pests, and to help mitigate the threats posed by emerging forest diseases^42^. In particular, drawing detailed maps of tree species that are capable of hosting harmful pathogens could provide an important resource in the context of pest-spread modelling and management^43,44^. Moreover, EU-Forest could be used in conservation management to accurately map the European distribution of tree species richness and rarity (Fig. 3) and improving our understanding of European tree biogeography (Fig. 4). For example, the dataset has already been used to show how the ecological spatial structure (in terms of nestedness) of actual tree vegetation departs from its natural potential^45^.

Knowing the distribution of forest tree species is also crucial to support ecosystem services and functions. A common assumption is that these aspects are closely associated to tree diversity, i.e., the more tree species a forest hosts, the higher its overall biodiversity, and the more numerous the ecosystem services it can offer^46^. This idea, which has clear implications for prioritisation and management, has been confirmed by large-scale studies in North America and part of China^47^. Nevertheless, it still lacks of convincing support in Europe, mostly due to limitations in the extension and resolution of previously available forest datasets that could not provide comprehensive coverage of diverse climatic and vegetation regions^7^. The high data density of EU-Forest may provide essential information to fill this knowledge gap.

Besides these general considerations, we are confident that the data we share in this paper will serve many other purposes. For instance, the EU-Forest dataset will also be extremely valuable in other disciplines such as palaeoecology and palaeoclimatology, improving reconstructions of past climate changes, and enabling interesting comparisons between the actual and palaeo-distribution of major tree species in Europe^48,49^.

## Methods

We merged information from the two largest existing datasets on European tree distribution (the Forest Focus and Biosoil databases, see previous paragraph), and an unpublished, much larger dataset derived from National Forest Inventories (hereafter, the ‘NFI dataset’). The NFI dataset was generated for the provision of forest data and services to support the European Forest Data Centre (EFDAC). This is the result of a framework contract established between the JRC of the European Commission (https://ec.europa.eu/jrc/) and European countries. The collection of tree occurrence data involved 19 EU Member States and two neighbouring countries (Norway and Switzerland), for a total of 21 countries^34^. Harmonisation procedures were agreed by the JRC and participant countries. These included the establishment of a common nomenclature for forest tree species among participant countries for the most common trees (approximately 200 tree species), and the establishment of standardised procedures for the provision of data to the JRC.

Although raw data uploaded by each country were available at a higher spatial resolution, they were subsequently aggregated at a lower spatial resolution of 1 square kilometre, in line with an INSPIRE-compliant 1 km×1 km grid^50^, specifically designed for pan-European mapping^51^. This upscaling procedure was necessary both for practical and legal reasons. First, it provided a way to standardise the information from different NFIs, which was heterogeneous in terms of spatial resolution and accuracy due to national differences in sampling design and in the establishment of sampling plots^52,53^. Second, the upscaling was a necessary condition to comply with different national rules regarding owners’ privacy protection, which often prevented the exact location of tree occurrences from being made public. For the same reason, the original data from Forest Focus and Biosoil, although available at higher than 1-km resolution, were harmonised with NFI data by attributing each occurrence record to the centroid of the corresponding cell into which the record fell.

The resulting EU-Forest dataset (Figs 1 and 2) includes a total of 1,000,525 occurrence records, 96% of which were obtained from the NFI dataset, with the remaining 4% coming from Biosoil and Forest Focus data. The NFI dataset occurrences are uniformly distributed over 248,776 plots across most of the European territory, although there are (few) geographical gaps for Poland, Croatia, Slovenia, Greece, Bulgaria, Cyprus, Belarus, Moldova and the Canary Islands (Fig. 2). The Forest Focus and Biosoil data are crucial to fill in some of these gaps with the addition of 8,564 plots and 20,634 occurrences from the former dataset, and 3,367 plots and 19,114 occurrences from the latter. Besides geographic extent, the NFI dataset is also much more complete than the other two datasets in terms of taxonomic diversity, including information for 78 tree genera and 242 tree species (c.f. Forest Focus: 23 genera and 47 species, and Biosoil: 57 genera and 187 species).

After the removal of incomplete records (i.e., where taxonomic identification was not at the species level) and of duplicate occurrences of the same tree species in the same plot (mostly arising from repeated sampling at different times), we obtained two datasets including over 249,410 plots, one at species level (588,983 occurrences), and one at genus level (589,657 occurrences). For the sake of completeness, we provide a summary table that summarizes the number of plots, the number of species/genera, and the number of species/genera occurrences per country (Table 1).

## Data Records

We provide two datasets for tree occurrences at, respectively, species and genus level (EU-Forest_species, EU-Forest_genus). Both datasets are available from *figshare* (Data Citation 1) as compressed (.zip) files in comma separated values (.csv) format, with 10 columns indicating coordinates in a ETRS89-LAEA reference coordinate system, representing the centroid of the INSPIRE-compliant 1 km×1 km European grid (X, Y); the country where the forest plot was sampled (COUNTRY); the source datasets (NFI, BS, FF); the name of the species/genus sampled (SPECIES/GENUS NAME); the class for the diameter at breast height (DBH-1, DBH-2) of the trunk, which is used to distinguish a new recruit of a given tree. This latter measurement has a value of 1 for trunks with a diameter of less than 120 mm, 2 for trunks with diameter greater than 120 mm, and −9999 for trunks with unknown diameters. The value of 120 mm represents the common minimum threshold adopted by the NFIs, although single NFIs have different minimum thresholds, ranging from 0 mm in Finland to 120 mm in Cyprus and Switzerland^54^. The choice of threshold has important implications, since estimations of biomass based on different thresholds can deliver very different results^55^.

In addition, for the species-level dataset, we provide an additional field stating whether or not the target occurrence falls within the species’ geographical range, measured as the extent of occurrence (EOO, see Technical validation paragraph for details). Finally, we provide two compressed archives (.zip) containing, respectively, the individual species occurrences as 242 point shapefiles, and the EOOs of all species having at least three occurrences as 203 polygon shapefiles.

## Technical Validation

We validated the scientific names of trees using the Taxonomic Name Resolution Service v4.0 (TNRS)^56,57^, replacing invalid synonyms with their valid names (e.g., *Acacia farnesiana* was replaced with *Vachellia farnesiana*). For the few records for which information was missing in the TNRS, we referred to the Integrated Taxonomic Information System (ITIS) Catalogue of Life^58^.

Although we obviously had no direct control of the raw tree occurrence data collected by individual countries, the fact that all surveys were conducted by trained professional staff using standardised protocols^52^ ensures data reliability. Nevertheless, we evaluated the overall meaningfulness of the dataset by comparing its biogeographical consistency with established knowledge. For this, we used a recent method based on network analysis^59^ to identify biogeographical regions for tree species in Europe (Fig. 4, left panel), applying the online tool Infomap Bioregions^60^ (http://bioregions.mapequation.org) at a spatial resolution of 0.5 degrees. This revealed a strong consistency with the biogeographical regions presented in Rueda *et al.*^61^ (Fig. 4, central panel) that, similarly to our approach, are based exclusively on tree species data. However, our results are also consistent with other classifications that take into account other variables such as climate, soil, and land cover^62–64^. The main differences between our classification and that of the European Environment Agency (EEA, latest version^62^) can be observed in Central-Eastern Europe and Fennoscandia (Fig. 4, right panel), where we could not distinguish between boreal and hemiboreal regions, probably due to the high presence of coniferous taxa in the latter. However, similarly to our classification, the EEA biogeographical regions show a distinct transition from West to East that is lacking from Rueda *et al.*^61^ map. Interestingly, the EU-Forest data led to the identification of three clearly distinct biogeographical Mediterranean regions (Iberia, Italy and Cyprus), and of an additional region in the British Isles. These distinctions, although absent from the EEA classification, are consistent with the independent analyses carried out by Casalegno *et al.*^16^, Barbati *et al.*^63^ and Metzger *et al.*^64^ The overall good fit between our biogeographical classification and previous studies provides indirect support to the soundness of our dataset, and highlights its potential for biogeographical studies and species distribution modelling.

We performed an additional validation by assessing the range (in terms of EOO) of each species that had at least three occurrences as alpha (α) shapes. This technique helps to identify the EEO of species with different degrees of restrictiveness, modulated by a single parameter (α). Large α values lead to EOOs close to the convex hull defined by the target species’ occurrences (in a two-dimensional space, given a set of points, the convex hull is the smallest convex polygon containing all points), while small α values generate EOOs close to the original set of points. Note that an EOO identified by the α-shape procedure may consist of disjointed polygons (this will be most likely the case for small α values), and may or may not include all of the occurrences.

We used an alpha level of 6 degrees, as recommended by Garcia-Rosello *et al.*^65^, using the ModestR software^66^. We then identified as possible outliers the occurrences falling outside the EOOs. Estimating EOOs using concave hulls is recommended over the standard use of convex hulls, because the former can better approximate species range by excluding discontinuities^67^. Following this analysis, we associated a binary field with each record in the dataset, indicating whether or not the target record fell within the corresponding species’ EOO. The EOOs for each species are provided as individual shapefiles (Data Citation 1).

## Additional Information

**How to cite this article:** Mauri, A. *et al.* EU-Forest, a high-resolution tree occurrence dataset for Europe. *Sci. Data* 4:160123 doi: 10.1038/sdata.2016.123 (2017).

**Publisher’s note:** Springer Nature remains neutral with regard to jurisdictional claims in published maps and institutional affiliations.

## Supplementary Material



## Figures and Tables

**Table 1:** Summary table illustrating the number of plots (N. Plots), number of species and their occurrences (N. Species and Species Occs.), number of genera and their occurrences (N. Genus and Genus Occs.) for each country listed in our dataset.

**Country**	**N. Plots**	**N. Species**	**Species Occs.**	**N. Genus**	**Genus Occs.**
Austria	3,525	66	10,924	34	10,816
Belarus	408	12	797	11	795
Belgium	32	24	108	20	104
Bulgaria	220	10	243	8	231
Croatia	78	25	181	16	179
Cyprus	19	14	71	9	50
Czech Republic	10,444	75	31,783	35	31,118
Denmark	2,470	47	7,208	32	9,185
Estonia	2,958	19	8,076	19	10,182
Finland	24,649	34	66,849	18	63,074
France	31,925	141	81,300	61	77,944
Germany	18,903	84	67,297	38	68,469
Greece	82	19	116	9	104
Hungary	4,428	81	15,427	39	13,076
Ireland	1,457	48	2,647	31	2,762
Italy	6,810	128	21,151	59	20,025
Latvia	4,144	28	5,701	21	8,444
Lithuania	2,093	34	7,454	18	8,786
Moldova	10	8	17	6	17
Netherlands	2,672	26	5,694	20	7,017
Norway	10,844	22	24,388	20	25,288
Poland	981	61	3,622	30	3,454
Portugal	4,646	17	6,211	17	7,261
Romania	3,291	92	13,814	46	13,138
Slovakia	1,449	64	4,311	33	4,122
Slovenia	69	64	558	32	500
Spain	74,411	130	119,360	64	111,065
Sweden	11,344	23	24,837	18	29,708
Switzerland	5,530	45	13,130	29	14,070
United Kingdom	19,518	52	42,949	32	49,793
The values are summarized after the technical validation (i.e., excluding occurrences falling outside of the respective alpha shape EOOs).					
